# Study on Properties and Optimization of Ternary Auxiliary Cementing Materials for IOTs

**DOI:** 10.3390/ma15113851

**Published:** 2022-05-27

**Authors:** Yannian Zhang, Xiangkun Zhang, Xiaowei Gu, Ting Wang, Bonan Liu

**Affiliations:** 1 School of Civil Engineering, Shenyang Jianzhu University, Shenyang 110168, China; zyntiger@163.com (Y.Z.); zxk1102286418@163.com (X.Z.); w18840653805@163.com (T.W.); 2 Science and Technology Innovation Center of Smart Water and Resource Environment, Northeastern University, Shenyang 110819, China; guxiaowei@mail.neu.edu.cn

**Keywords:** iron tailing, supplementary cementitious materials, compressive strength, microstructure

## Abstract

In order to control energy consumption and reduce pollution, the use of supplementary cementitious materials (SCMs) instead of cement to produce green cementitious materials can save energy, reduce emissions and achieve sustainable development. This study demonstrates the possibility of developing SCMs with iron tailings (IOTs), fly ash (FA) and ceramic powder (CP) ternary system, as well as the optimization and improvement scheme of gelation activation. The effects of activator dosage, mix ratio and substitution rate on mechanical properties of ternary SCMs system were investigated. The formation and evolution of hydration products were analyzed by differential thermogravimetric analysis (DTA) and scanning electron microscopy (SEM). The results of the study show that there is synergy in the system. The results show that there is synergy in the system and the hydration reaction is sufficient. At the substitution rate of 30%, the doping ratio of IOTs, CP and FA is 1:2:2 and the Ca(OH)_2_ is 0.6%, the strength reaches 39.9 MPa and the activity index is 91.5%, which can provide a basis for the application and more in-depth study of IOTs multi SCMs.

## 1. Introduction

With the continuous promotion of global infrastructure construction, cement and concrete as the main building materials, its demand is increasing, and with it comes the problem of serious environmental pollution and increasing energy consumption. Therefore, the cement and concrete industry needs to adjust the development mode, change the direction of development, reduce energy consumption, reduce environmental pollution, and take the road of sustainable development [1]. The decisive material in cement and concrete is the cementitious material, and the traditional cementitious material is usually cement-based, fly ash, mineral powder, silica fume and other industrial by-products as mineral admixtures [2,3,4,5]. The construction industry consumes a large amount of steel and cement, especially in the process of cement production, which emits gases such as CO_2_, SO_2_, SO_3_ and NO_2_ that cause serious pollution to the environment. According to statistics, each ton of cement produced emits about one ton of gases, and reducing the production of cement will make an important contribution to reducing emissions. For this reason, the EU government has committed to reduce 30% of the gases affecting the greenhouse effect by 2020 and 95% by 2050 [6]. Use the advantages of the cement industry to effectively absorb waste, increase the resource utilization of various solid wastes, achieve low pollution and low emissions in the cement industry, and promote the cement industry to become a circular economy industrial system with coordinated and sustainable development of resources, environment and human society [7,8].

The role of industrial waste, mining solid waste and municipal waste as mineral admixtures in cementitious materials to control energy consumption and reduce environmental pollution is becoming more and more prominent. The accumulated solid waste not only causes waste of resources, along with the waste of land resources and serious pollution of the environment, endangering the ecosystem. Mineral admixture can not only alleviate the problem of energy consumption, but can also play a positive role in protecting the environment, and is the road to sustainable development of today’s green high-performance concrete [9,10,11]. Fly ash, silica fume and mineral powder, as common mineral admixtures, are severely limited in their capacity due to energy saving and emission reduction requirements [12,13,14]. Therefore, we need to find new mineral admixtures to meet the market demand and follow the trend of energy saving and emission reduction and green development.

Iron tailings (IOTs) are mining solid wastes, and there are a large amount of IOTs produced every year in China. The accumulation of a large amount of IOTs wastes land resources and pollutes the environment. Additionally, IOTs are potential secondary resources, besides containing valuable metals, vein minerals and other materials are the main mineral composition of IOTs, and oxides of silicon, calcium and aluminum are the main chemical components. However, IOTs are inert materials that do not have high cementing activity [15]. To effectively utilize IOTs, they must first be activated, thus stimulating the activity. Single-doped IOTS has a negative impact on compressive strength [16]. Over the years, many scholars have conducted various studies on IOTs as SCMs. Zhao et al. [17] investigated the effects of particle size and conditioning temperature on the properties of IOTs-doped cement slurry and found that partial replacement of cement by IOTs accelerated the hydration of cement at the initial stage. Yang et al. [18] used IOTs as concrete admixture to study the activity optimization of cementitious materials and found that mechanical activation was an effective method to improve the activity of IOTs. Han et al. [19] studied the early hydration characteristics of composite binder containing IOTs powder and found that the early hydration of IOTs was extremely low, but the gradation of tailings and cement particles was good, and the compressive strength of IOTs was not significantly reduced by increasing the admixture of IOTs. Yao Lei et al. [20] prepared C40 concrete with compressive strength higher than normal concrete by using IOTs as raw material after grade optimization of natural fine sand, artificial coarse sand and IOTs. However, in these studies, the activity index of the activated IOTs still did not meet the standards for blending [21], and the tailings blending was low.

China is the largest producer of ceramics in the world and produces about 10 million tons of ceramic (CP) waste each year, which leads to a serious waste of land resources and environmental pollution. The raw materials of ceramic products are clay, quartz and feldspar, which are then fired at high temperature to form a CP after grinding [22]. Others [23] have shown by the test, after grinding to a certain degree of fineness of the CP has obvious volcanic ash effect, if at room temperature, moisture and with a certain degree of alkali environmental conditions, the later can be with Ca(OH)_2_ secondary hydration reaction, thus generating a large number of C-S-H and C-A-H and other substances with a certain degree of cementation, and hardening to produce strength, and it has been demonstrated that CP has no significant positive effect on the early strength development of cement mortar. It mainly acts as a microfiller, resulting in a low early strength of cement mortar. However, due to the volcanic ash activity of CP [24], the late strength development of concrete incorporated with CP was enhanced.

Fly ash (FA) is the tiny fine ash particles collected from the flue gas produced by burning coal, and is the main solid waste from coal-fired power plants. The use of FA mixed into the original concrete to replace part of the cement can optimize the performance of concrete and improve the strength of concrete, because FA can not only save the amount of raw materials, but also can play the “volcanic ash effect” and “morphological effect” and “micro-aggregate effect”. In general, FA is similar to CP in that it is less efficient in the initial stage of cementation and acts more as a microfiller, but the volcanic ash properties prove to be effective in later stages, leading to a significant increase in strength [25]. It has been documented that FA has certain volcanic ash reactivity, and when it is mixed into cement as admixture, its active components SiO_2_ and Al_2_O_3_ can react with Ca(OH)_2_ produced during the hydration of cement clinker to produce hydration products such as C-S-H and C-A-H [26], which is one of the main bases for FA to function in cement and concrete, respectively. Another study [27] pointed out that FA play a “morphological effect” and “micro-aggregate effect” can play a lubricating role in the material, filling the gaps in the concrete to increase the density of concrete and improve the working properties of concrete.

IOTs, CP, and FA are three materials used as SCM and have been the subject of a lot of research, but still cannot achieve a large consumption of solid waste, and there are many problems. It has been pointed out that there is a synergy between different materials that can be used to adjust the working properties, mechanical properties and particle gradation of concrete through multiple components [5]. This synergistic effect combining these multiple components together is more effective than using them individually. CP and FA are aluminum-rich waste, in the system of secondary hydration can produce C-A-S-H, so that the hydration products are more abundant, enhance the density of the matrix, and IOTs can fill in the pores to further improve the density, and theoretically should be able to achieve the synergistic purpose.

In this study, we study the ternary SCMs, analyze the co-hydration process of IOTs, CP, FA and cement, clarify the physical phase changes, microscopic morphological structure evolution and macroscopic mechanical property development law during the hydration process, and elucidate the effects of multi-solid waste characteristics on the hydration process and hydration mechanism of the SCM system.

## 2. Materials and Methods

### 2.1. Raw Materials

The experimental materials included IOTs, CP, FA, Portland cement, standard sand, and water. Ca(OH)_2_ (The purity > 99%) was used as chemical activator. The IOTs in this study were obtained from Wai tou Mountain, Benxi City, Liaoning Province, China. The FA was produced in Ya tai Company, Liaoning Province, China. The CP used is provided by Chaozhou City, Guangdong Province, Xin huan Technology Co. The chemical compositions of the three types of materials are shown in Table 1. The cement used in this test comes from Shan shui Gong yuan Cement Co., Ltd. and is 425 ordinary silicate cement (hereinafter indicated by the symbol “P-O 42.5”), the performance of which conforms to the requirements of GB175-2007 “General Silicate Cement”.

**Figure 1:**
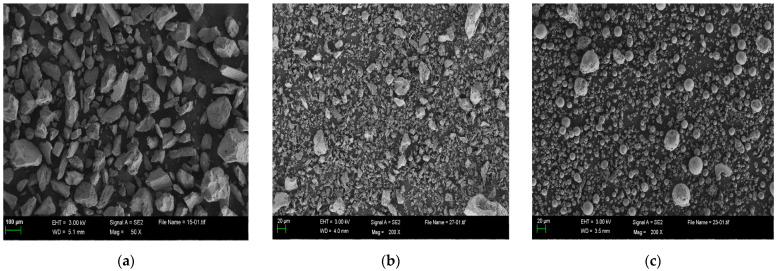
SEM image of (**a**) IOTs; (**b**) CP; and (**c**) FA.

**Figure 2:**
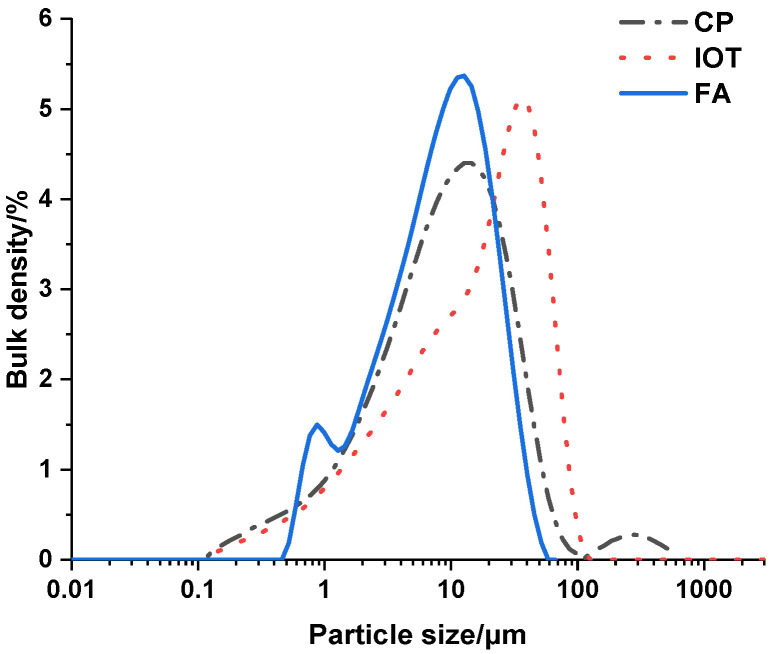
Particle size distribution of IOTs, CP and FA.

The microscopic characterization and the particle morphology were measured via BET and SEM (see Figure 1). The specific surface area and particle size distribution of IOTs, CP and FA are shown in Table 2 and Figure 2.

### 2.2. Mix Design and Specimens Preparation

Preparation of mineral admixture by compounding IOTs, CP and FA. According to the Chinese national standard GB/T12957-2005 [28] Industrial Waste Residue Activity Test Method for Cement Mixture and GB/T17671-1999 [29] Cement Mortar Strength Test Method (ISO Method), cement mortar was prepared, compressive strength test and activity index calculation were carried out.

Each material was weighed according to Table 3, Table 4 and Table 5. Where Table 3 indicates the Group A trials with different substitution rates as variables. Table 4 indicates the Group B trials with different proportioning as the variable. Table 5 shows the Group C tests with activator doping as a variable. Mix the raw materials in JJ-5 planetary mixer with 0.5 water-cement ratio, set the automatic mode after mixing, load the mortar into 40 mm × 40 mm × 160 mm triplex mold, maintain it in the standard maintenance room with constant temperature of 20 ± 1 °C and relative humidity of 95 ± 1% for 24 h and then demold it, and continue to place it in the maintenance room until the corresponding age.

The net paste is mixed and prepared in accordance with the test requirements of cement and the corresponding proportion of each admixture, then take the appropriate amount of mixed materials in disposable plastic cups and other containers, water to glue ratio of 0.5, add the appropriate amount of deionized water, while stirring with a glass rod and pour into the mold, and then in the temperature of 20 ± 1 °C, relative humidity of 95 ± 1% of the constant temperature and humidity standard curing room curing time of 24 h. After that, the mold was demolded and continued to be placed in the maintenance room until the corresponding age. After reaching the specified curing time, the core of the hydrated hardened paste and the mortar were taken out after drying at 50 °C for 2 h, and then soaked in anhydrous ethanol for 3 days to terminate the hydration. The purified slurry sample after the termination of hydration was dried at 50 °C for 24 h, followed by grinding to prepare the samples for thermogravimetric analyses. After cutting and taking the block, it was ground on sandpaper, and cured with epoxy resin for 24 h. After grinding the surface, it was put into isopropanol solution for ultrasonic cleaning to prepare SEM sample.

### 2.3. Experimental Procedures

#### 2.3.1. Compression Tests

Compression tests were performed via GYE-300B universal testing machine (Beijing Kodak Jinwei Technology Development Co., Ltd., Beijing, China). A loading rate of 2.4 kN/s, as stipulated by GB/T17671-1999, was conducted during the loading.

The activity index of 7 d and 28 d mortar was determined according to the national standard GB/T12957-2005. The activity index can be calculated according to the following equation:

K = R_1_/R_2_ × 100% (1)

where R_1_ is the compressive strength of the admixture system test sample at 7 d and 28 d. R_2_ is the compressive strength of the cement mortar for 7 d and 28 d.

#### 2.3.2. Thermogravimetric Analysis (DTA-TG)

DTA-TG was conducted using TA instruments (Q500 V20.13, TA Instruments, New Castle, DE, USA) in a nitrogen gas flow with a 70 mL/min flow rate. Each testing sample was about 15 mg. The TGA was exposed to the temperature from room temperature to 800 °C with a rate of 20 °C/min.

#### 2.3.3. Scanning Electron Microscopy (SEM)

The microstructure of the hydration products of the paste samples at 28 days was analyzed via SEM (Carl Zeiss AG, Oberkochen, Germany). All specimens were immersed in anhydrous ethanol for 3 days before the SEM test to prevent water and reaction, and then the samples were dried in the oven to remove anhydrous ethanol.

## 3. Results and Discussion

### 3.1. Compressive Strength Analysis

#### 3.1.1. Compressive Strength Analysis with Different Substitution Rates

**Figure 3:**
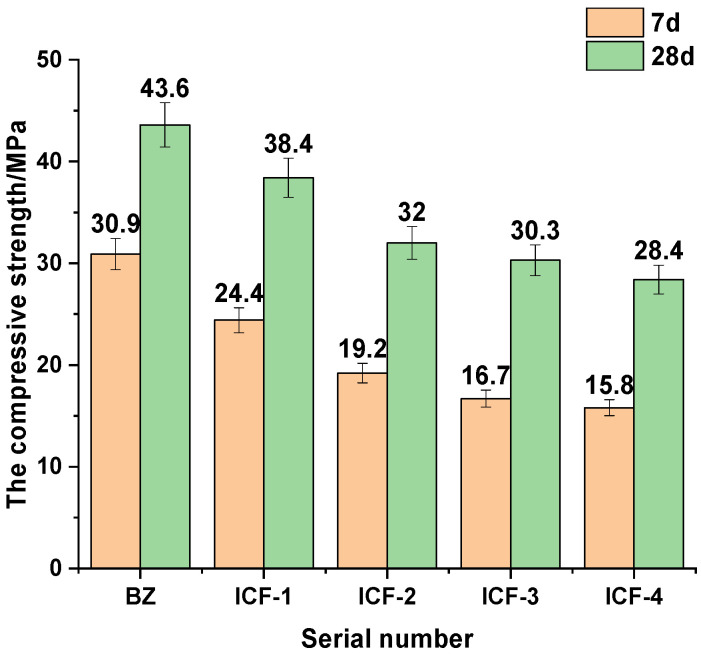
Compressive strength tests with different substitution rates.

Figure 3 shows the effect of different substitution rates on the compressive strength of the SCM system at different ages of 7 d and 28 d. It can be seen that the compressive strength is decreasing with the increase of ternary dopant substitution rate. The reason is that IOTs, CP and FA do not have potential hydraulic hardness in the early stage of hydration, and some volcanic ash reactions exist but are very weak, and most of them play a filling role [19,23,25]; in the late stage of hydration reaction, the active SiO_2_ and Al_2_O_3_ and Ca(OH)_2_ in the SCM system continuously undergo secondary hydration reactions to generate hydration products such as C-S-H, C-A-H and AFt, but due to the reduction of cement, the amount of Ca(OH)_2_ that can undergo secondary hydration is small and the degree of secondary hydration is low, leading to an increase in substitution rate and a decrease in both strength and activity index. After ICF-2, the decrease of compressive strength decreases, which means that the influence of the change of substitution rate on the compressive strength of the system has been weakened, and the comprehensive consideration is that the 7 d compressive strength of ICF-1 with 30% substitution rate is 24.4 MPa, the activity index is 79%, the 28 d compressive strength is 38.4 MPa, the activity index is 88.1% can meet the requirements, and the fixed substitution rate is 30% is more suitable. The fixed replacement rate is 30%.

#### 3.1.2. Compressive Strength Analysis under Different Proportioning

**Figure 4:**
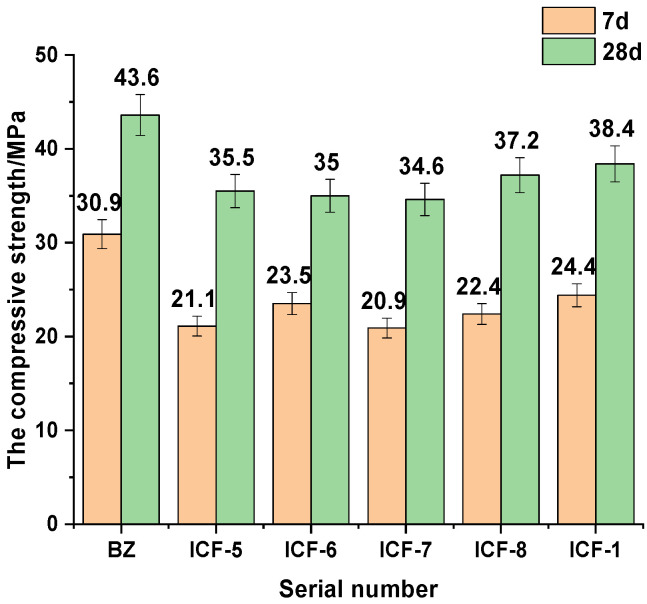
Compressive strength tests with different proportioning.

Figure 4 shows the effect of different composition ratios on the compressive strength of the system at different ages of 7 d and 28 d. It is seen from the graph that the compressive strength and activity index are decreasing with increasing CP dosing and decreasing FA. The reason lies in the fact that FA volcanicity is higher than CP and IOTs [18,30,31], and the high degree of secondary hydration with a large proportion of FA, which in turn can produce more hydration products such as C-S-H, C-A-H and AFt to improve the strength. Interestingly, a clear inflection point appears at ICF-6. The reason for this is that when the CP to FA ratio is 1:3, the CP doping is low and the filling effect is not obvious, while the FA doping is high and plays a more obvious filling effect, and the FA volcanic ash activity is higher than that of CP, with more volcanic ash active fraction [30]. When the ratio of CP to FA is 2:2, at this time, the CP doping increases and reaches a good gradation with FA, the filling effect is obvious, and part of CP can be used as an additional nucleus to promote hydration [32,33], and CP has the largest specific surface area in the SCM system, which can accelerate the hydration process of cement and generate more hydration products gel to fill the matrix and improve the density of the matrix so that the strength has a certain rise. When the ratio is 3:1, the amount of CP is too large, because its volcanic ash activity is lower than that of FA, at this time the system can participate in the hydration of less material, the degree of hydration is low, the favorable effect of CP filling and larger specific surface area on the matrix is lower than the adverse effect of the reduction of volcanic ash active material, thus the compressive strength and activity index have significantly decreased. Therefore, CP and FA should be fixed in the ratio of 2:2 to ensure the compressive strength and activity.

#### 3.1.3. Compressive Strength Analysis of Activator Doping

**Figure 5:**
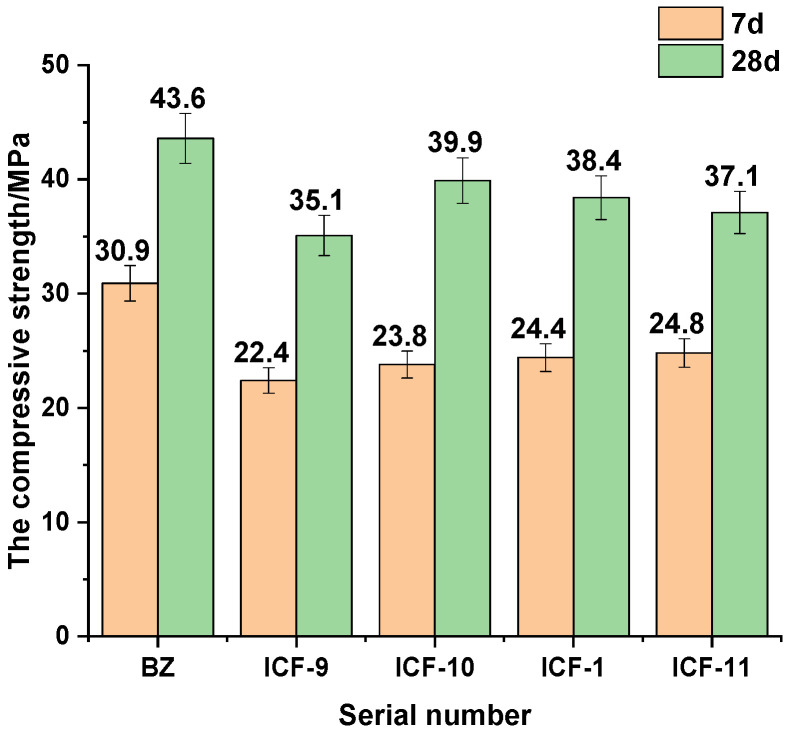
Compressive strength tests with different activator doping.

Figure 5 shows the effect of Ca(OH)_2_ dosing on the compressive strength of the system at different ages of 7 d and 28 d. It can be seen the effect of Ca(OH)_2_ doping on the compressive strength at different ages showed different trends. At the age of 7 d, the compressive strength increased with the increase of Ca(OH)_2_ dosing; while at the age of 28 d, the compressive strength showed a trend of increasing and then decreasing with the increase of Ca(OH)_2_ dosing. The reason [34] is that Ca(OH)_2_ added at the early stage of hydration reaction will release Ca^2+^ and OH^-^ microsolubly, and under the action of OH^-^ ions, the Si-O and Al-O bonds with high polymerization in the SCM system will break and form unsaturated bonds, releasing a large number of reactive ions, and the system containing SO4^2-^ can generate AFt at the early stage of hydration to guarantee the early strength, so at 7 d time, the compressive strength increases continuously with the increase of Ca(OH)_2_ doping. When the hydration reaction is late, the Ca(OH)_2_ mixed with too much is fully dissolved, and some silicate and aluminate combine with Ca^2+^ to form C-S-H and C-A-H, which consume part of the active products, thus reducing the system activity, and the OH- content is too high, which inhibits the hydration reaction of cement [34], and AFt will be converted into AFm, and the overall strength decreases. In order to ensure the overall strength and activity of the specimen, it can be seen that it is appropriate to choose Ca(OH)_2_ doping amount of 0.6%.

### 3.2. DTA-TG Analysis

**Figure 6:**
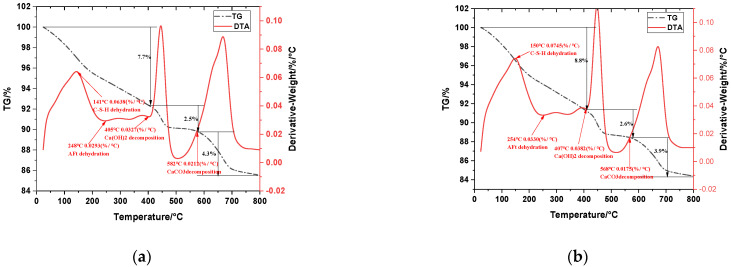
DTA-TG curves of ICF-10 group at the age of (**a**) 7 d and (**b**) 28 d.

As can be seen in Figure 6, the samples had four heat absorption peaks and four thermal weight losses at both 7 d and 28 d of hydration age. The C-S-H gel had a heat absorption peak at 140–150 °C, which was the result of dehydration of the C-S-H gel, and its dehydration amount was determined by the drying conditions of the sample before the test [35]. The heat absorption peak at around 250 °C indicates the dehydration of AFt. A large heat absorption peak at 400–450 °C indicates the dehydration decomposition of CH. A heat absorption peak at 600–700 °C indicates the decomposition of CaCO_3_ produced by the carbonization of CH [36].

In order to visualize the hydration process of the ternary SCM system, the CH content of each group of samples at different ages was calculated using Equations (2) and (3).

(2) $$CH=WL_{CH}\times \frac{m_{CH}}{m_{H_{2}O}}+WL_{CaCO_{3}}\times \frac{m_{CaCO_{3}}}{m_{CO_{2}}}$$

In the formula: *CH* is the relative content of calcium hydroxide in the sample (%); *WL_CH_* is the mass loss of calcium hydroxide caused by removing water through TG (%); *WL_CaCO_3__* is the mass loss of calcium carbonate caused by removing water through TG (%); *m_CH_* is the molar mass of calcium hydroxide; *m_H_2_O_* is the molar mass of water; *m_CaCO_3__* is the molar mass of calcium carbonate; and *m_CO_2__* is the is the molar mass of carbon dioxide. The values of *WL_CH_* and *WL_CaCO_3__* can be obtained by data processing through the TG curve. *m_CH_* = 74 g/mol; *m_H_2_O_* = 18 g/mol; *m_CaCO_3__* = 100 g/mol; and *m_CO_2__* = 44 g/mol. The molar masses of calcium hydroxide, water, calcium carbonate and carbon dioxide can be obtained by substituting them into Equation (2).

(3) $$CH=WL_{CH}\times \frac{74}{18}+WL_{CaCO_{3}}\times \frac{100}{44}$$

The calculated results are shown in Table 6. The content of CH in cement-based hardened slurry cementitious system is closely related to the degree of hydration of cement, and the higher the content of CH indicates the higher the degree of hydration [37]. It can be seen that the CH content of the ICF system decreases with the age of hydration because the introduction of admixtures reduces the amount of cement used, which in turn reduces the process of cement hydration and leads to a decrease in CH content, and the ICF admixture system participates in the secondary hydration reaction and thus consumes the CH generated by cement hydration in the system. This is consistent with the previous analysis of compressive strength.

### 3.3. SEM Analysis

**Figure 7:**
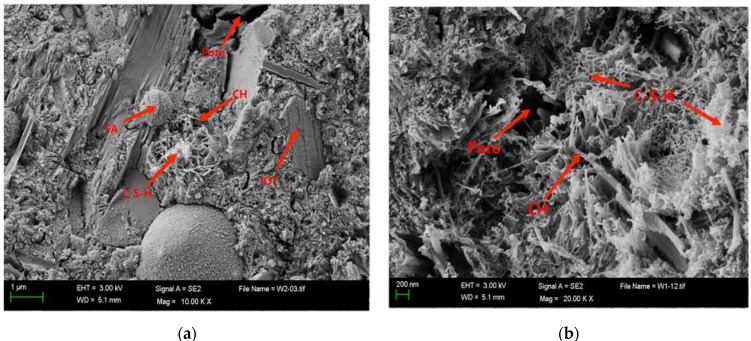
SEM analysis results of the ICF-10 specimen for 28 days. (**a**) Not fully hydrated; and (**b**) more fully hydrated.

Figure 7 shows the SEM microscopic morphology of ICF-10 group at 28 days, from Figure 7a it can be clearly seen that there are some hydration products, flocculent C-S-H gel, but the hydration products are not full, there are pores, and the bonding between some particles is relatively independent, especially as there are a large number of fluting irregular unhydrated IOTs. The volcanic ash activity of FA is significantly higher than that of CP and IOTs, which is consistent with the compressive strength, and the greater the FA dose, the higher the active part of volcanic ash, the higher the degree of participation in secondary hydration, and the higher the strength. Figure 7b shows that the hydration products are very abundant, and a large number of clustered and partially fibrous C-S-H gels can be observed on the particle surface, with tighter bonding between the hydration products. The fibrous C-S-H gels grew faster and started to lap each other to form a mesh structure [38]. In the system, IOTs, CP, and FA reacted with Ca(OH)_2_, the hydration product of residual cement particles, and the amount of C-S-H gels increased. Additionally, due to the different fineness of IOTs, CP, and FA, these residues are compounded together to fill each other and form a good gradation, and the particle size distribution of fine particles in the whole system is more reasonable and the pore structure is fully refined, which makes the strength increase significantly. In the figure, we can see many reacted IOTs particles, in synergy with CP and FA, IOTs not only play the role of micro-aggregate filling, it also participates in a large number of hydration reactions, and the three have good synergy. There is a synergistic hydration reaction of solids in the cementitious material system, which promotes the increase of the system strength [11].

## 4. Conclusions

In this paper, the effects of different influencing factors of activator dosing, composition ratio and substitution rate on the mechanical properties and microstructure of the ternary SCM system of IOTs, CP and FA were investigated. Based on the above experimental results and related discussions, the main conclusions are as follows:

(1) At the substitution rate of 30%, the ratio of IOTs, CP and FA is 1:2:2 and Ca(OH)_2_ is 0.6%, the strength reaches 39.9 MPa and the activity index is 91.5%. It has the potential to develop SCMs that can reduce cement usage, enable resourceful use of IOTs, CP and FA, and promote energy saving and green development.
(2) It can be seen in both macro and micro perspectives that FA volcanicity is higher than IOTs and CP. The FA in the system participates in the volcanic ash reaction, CP provides additional nuclei to accelerate the hydration process, and IOTs play a filling effect to provide nucleation sites, and the three materials hydrate synergistically.
(3) The amount of CH added had a significant effect on the compressive strength of the slurry. Overall, 0.6% CH promoted the formation of CSH gel, while when the amount of CH was too much, cement hydration was inhibited, and CaCO_3_ inhibited the growth of strength.
(4) Ternary SCMs system has no hydraulic property, only volcanic ash property. When the replacement rate is too high, it will seriously harm the compressive strength. It is recommended that the replacement rate be less than 30%.

In summary, the appropriate amount of IOTs, CPs, and FAs is expected to be a new SCM to reduce the cement dosage in terms of mechanical properties, microstructure, and co-hydration analysis. In addition, further studies on the degree of hydration of this SCM under the conditions of other activators and the mechanical properties and durability performance of concrete prepared with this SCM are necessary.

## Figures and Tables

**Table 1:** Chemical composition and content of materials (mass fraction/%).

	SiO_2_	Al_2_O_3_	Fe_2_O_3_	MgO	CaO	SO_3_
IOTs	62.26	4.78	14.37	6.33	7.77	0.48
CP	62.56	23.41	1.32	1.56	6.34	0.06
FA	60.10	25.10	6.74	0.86	2.93	0.27

**Table 2:** The specific surface area of the materials.

Materials	IOTs	CP	FA
Specific surface /m^2^.kg^−1^	1290	1898	1391

**Table 3:** Cement mortar ratio of group A.

Serial Number	Cement/g	IOTs/g	CP/g	FA/g	Standard Sand/g	Ca(OH)_2_/g
BZ	450	0	0	0	1350	-
ICF-1	315	27	54	54	1350	1.08
ICF-2	292.5	31.5	63	63	1350	1.08
ICF-3	270	36	72	72	1350	1.08
ICF-4	247.5	40.5	81	81	1350	1.08

**Table 4:** Cement mortar ratio of group B.

Serial Number	Cement/g	IOTs/g	CP/g	FA/g	Standard Sand/g	Ca(OH)_2_/g
BZ	450	0	0	0	1350	-
ICF-5	315	67.5	16.875	50.625	1350	1.08
ICF-6	315	67.5	33.75	33.75	1350	1.08
ICF-7	315	67.5	50.625	16.875	1350	1.08
ICF-8	315	45	45	45	1350	1.08

**Table 5:** Cement mortar ratio of group C.

Serial Number	Cement/g	IOTs/g	CP/g	FA/g	Standard Sand/g	Ca(OH)_2_/g
BZ	450	0	0	0	1350	-
ICF-9	315	27	54	54	1350	0
ICF-10	315	27	54	54	1350	0.81
ICF-11	315	27	54	54	1350	1.35

**Table 6:** TG test of the yield of each substance.

Serial Number	Age	CH Take off the Water	Amount of CaCO_3_ Decomposition	C-S-H Decomposition Quantity	CH Content
ICF-10	7 days	2.5%	4.3%	7.7%	20.1%
ICF-10	28 days	2.6%	3.9%	8.8%	19.5%

## Data Availability

All data is provided in the manuscript.
